# Biphasic Dose–Response and Mechanism Analysis of Vanillic Acid from *Larix gmelinii* on *Neofusicoccum laricinum*

**DOI:** 10.3390/ijms27125159

**Published:** 2026-06-06

**Authors:** Xinyan Chen, Ruizhi Zhang, Zhiyang Zhang, Haoru Wang, Yizhen Zhang, Danlei Li, Feng Wang

**Affiliations:** 1 Key Laboratory of Alien Forest Pest Detection and Control-Heilongjiang Province, School of Forestry, Northeast Forestry University, Harbin 150040, China; cxy0201@nefu.edu.cn (X.C.); zhangruizhi@nefu.edu.cn (R.Z.); wanghaoru506@163.com (H.W.); 15539883952@163.com (Y.Z.); 2 Key Laboratory of Sustainable Forest Ecosystem Management-Ministry of Education, Northeast Forestry University, Harbin 150040, China; zy_zhang0314@163.com; 3 State Key Laboratory of Tree Genetics and Breeding, School of Forestry, Northeast Forestry University, Harbin 150040, China

**Keywords:** larch shoot blight, phenolic acid, metabolomics, transcriptomics, phenolic acid degradation pathway

## Abstract

Phenolic levels in *Larix gmelinii* are closely correlated with its interaction with *Neofusicoccum laricinum*. As a vital subclass of phenolics, phenolic acids participate in both constitutive and inducible plant defense responses. In the present study, metabolomic, transcriptomic, physiological and biochemical analyses were conducted to investigate the tissue heterogeneity of phenolic acid metabolism in larch stem tissues, as well as the dose-dependent effects and underlying mechanism of vanillic acid (VA) on *N. laricinum*, providing valuable insights into the interaction between *L. gmelinii* and *N. laricinum*. A total of 119 differentially accumulated phenolic acid metabolites were identified in this study. Shoots exhibited markedly higher levels of most phenolic acids, including VA, than trunks and branches, accompanied by significant enrichment of the phenylpropanoid biosynthesis pathway. Notably, VA exhibited a biphasic dose–response on *N. laricinum*, facilitating the growth of *N. laricinum* at low concentrations (2–1117.12 μg/mL) and exerting inhibitory effects at high concentrations (>1117.12 μg/mL). Transcriptomic analysis further suggested that phenolic acid metabolism and the overcompensation effect collectively accounted for this unique dose–response pattern. Collectively, this study verified that phenolic acid metabolism across *L. gmelinii* stem tissues presented distinct tissue heterogeneity, and VA exerted a biphasic dose–response on *N. laricinum*.

## 1. Introduction

Larch shoot blight is a devastating disease caused by the invasive fungal pathogen *Neofusicoccum laricinum* [1,2,3]. Dominant native larch species in China, such as *Larix gmelinii*, are all susceptible to this disease [4]. It broke out and became epidemic in larch forests across China, spreading across eight provinces and 110 counties (cities) by 1992, becoming a critical limiting factor for the healthy growth of larch forests and ecological sustainability [5]. However, research on larch shoot blight has been limited in recent years. There is still a lack of research on the molecular mechanisms underlying the interaction between larch and *N. laricinum*.

Plants possess multiple defense systems against phytopathogens, among which secondary metabolism is essential for responding to fungal infection and building basal defenses [6]. *N. laricinum*, a common stem pathogen of larch, preferentially infects shoots. It can persist latently in perennial branches and trigger branch dieback, but trunk infection is rarely reported [7,8,9]. Larch shoots, branches and trunks (hereafter collectively referred to as stem tissues) differ markedly in their susceptibility to *N. laricinum*, and tissue lignification has been proposed as a key cause for this variation [4,5,7]. Plant disease resistance is, however, governed by multiple pathways. Basal metabolism in healthy tissues also shapes host resistance and affects pathogen infection and colonization. The regulatory role of basal metabolism in the larch–*N. laricinum* interaction still awaits in-depth exploration.

Previous studies have shown that basal levels of endogenous phenolics are strongly associated with larch shoot blight, highlighting the vital function of phenolic metabolism in the larch–*N. laricinum* interaction [10]. Phenolic acids, a major subclass of phenolics, are ubiquitous and stably present in plant tissues and contribute to both constitutive and inducible plant defenses [11,12]. According to their chemical structures, they are mainly categorized into benzoic acid-type, phenylacetic acid-type, cinnamic acid-type compounds and phenolic acid derivatives. Multiple studies have verified the antimicrobial activity of phenolic acids against plant pathogens. For instance, vanillic acid (VA), protocatechuic acid (PA) and chlorogenic acid have been proven to suppress multiple phytopathogenic fungi [13,14,15,16,17,18,19]. These results suggest that phenolic acid metabolism may mediate the interplay between larch and *N. laricinum*. However, phenolic acid profiles and concentrations vary markedly among plant tissues, and different monomers exert differential effects on pathogens [20]. The biological actions of exogenous compounds against organisms are generally interpreted via dose–response relationships, which can be categorized into monophasic, biphasic and triphasic dose responses [21]. Studies have shown that phenolic acids may exert a biphasic dose effect on pathogenic fungi, which is commonly observed in *Fusarium oxysporum* [22]. Currently, studies on larch phenolic acids remain limited. As the major tissues where *N. laricinum* infects and persists latently, larch stem tissues have not been thoroughly investigated regarding the tissue heterogeneity of phenolic acid metabolism and the actual effects of phenolic acids on *N. laricinum* [23,24]. This lack of basic data impedes further exploration of the regulatory mechanisms underlying the larch–*N. laricinum* interaction.

As analytical techniques evolve rapidly, omics approaches have been widely used to clarify the tissue heterogeneity of metabolic profiles and associated molecular mechanisms in plants [25,26,27,28]. In the present study, untargeted metabolomics was applied to profile phenolic acids in distinct stem tissues of healthy *L*. *gmelinii* and characterize their metabolic differences and tissue heterogeneity. By taking VA as the research object and combining model fitting with transcriptomic analysis, this study further analyzed the dose–response relationship of phenolic acids on *N. laricinum* and the underlying molecular mechanisms.

This study clarified the tissue heterogeneity of phenolic acid metabolism within stem tissues of *L. gmelinii* and revealed the biphasic dose–response effect and underlying molecular mechanisms of VA on *N. laricinum*. These findings provide a preliminary basis for elucidating the regulatory roles of phenolic acids during the interaction between *L. gmelinii* and *N. laricinum*, as well as a dataset for the differential phenolic acid metabolism in *L. gmelinii* stem tissues. Furthermore, this work offers insights into the development of environmentally friendly strategies for integrated management of larch shoot blight.

## 2. Results

### 2.1. Confirmation of Healthy Larch Forest Stands and Samples

**Figure 1:**
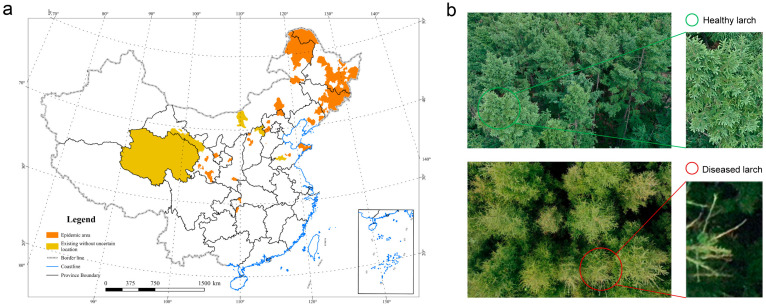
Distribution and Field Investigation of Larch Shoot Blight. (**a**) Distribution of larch shoot blight; (**b**) drone imagery of diseased and healthy larch stands.

According to the statistical investigation, larch shoot blight has been reported in 142 counties, 49 cities, and 13 provinces in China (Figure S1), with no reported revocation of disease occurrence areas involving 121 counties in 13 provinces (Figure 1a). Given that larch shoot blight had been documented in Harbin, Heilongjiang Province, drone surveys were carried out to identify relatively healthy larch stands. Image comparison analyses verified that drone imagery could clearly distinguish healthy stands from diseased ones. Symptomatic stands infected with larch shoot blight exhibited typical disease features, including needle yellowing and defoliation, whereas healthy plantations maintained vibrant green and intact needles (Figure 1b). Healthy individual trees in the healthy stands were thus selected for subsequent experiments.

### 2.2. Analysis of Metabolic Differences in Stem Tissues of L. gmelinii

#### 2.2.1. Untargeted Metabolomics Analysis

**Figure 2:**
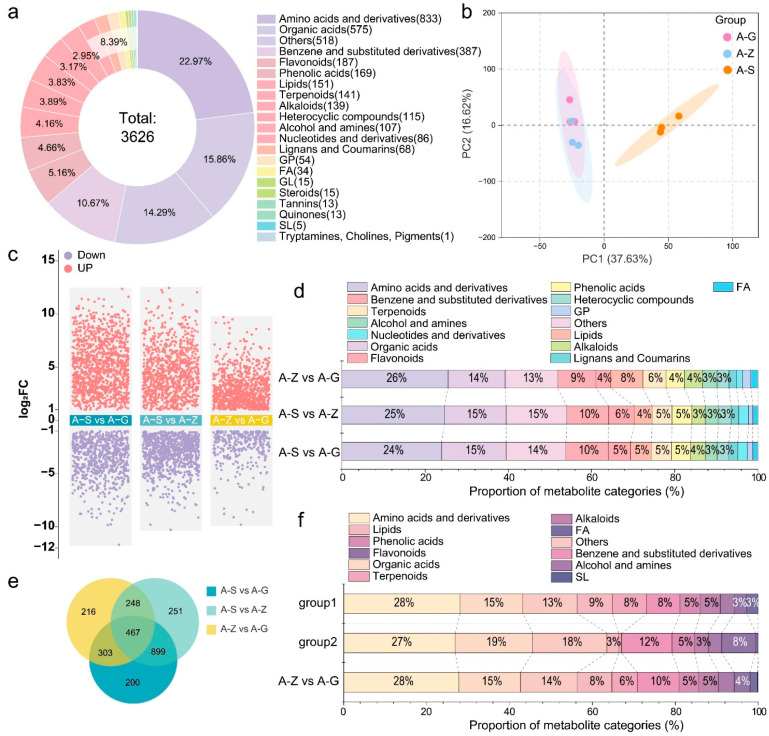
Untargeted metabolomic analysis of shoots, branches and trunks of *L. gmelinii*. (**a**) Classes of metabolites identified by untargeted metabolomic analysis; (**b**) PCA of samples (shaded colored ellipses denote 95% confidence ellipses for each group); (**c**) volcano plots of differential metabolites (DMs) screened using variable importance in projection (VIP) > 1 and |log_2_ fold change (FC)| > 1 among three comparison groups (A-S vs. A-Z, A-S vs. A-G, and A-Z vs. A-G); (**d**) analysis of the relative proportions of DM classes among different comparison groups; (**e**) Venn analysis of DMs among different comparison groups; (**f**) analysis of the relative proportions of major DM classes in group 1, group 2, and A-Z vs. A-G. The SL substances segment only appears at the bottom of the A-Z VS A-G bar with a very low proportion, and is absent in Group 1 and Group 2.

Prior to analysis, the consistent peak profiles and TIC distributions across test and QC samples confirmed the stability and reproducibility of the entire analytical workflow (Figure S2). After quality control (QC), a total of 3626 metabolites were identified across all samples, among which 1534 were detected in positive ion mode and 2092 in negative ion mode. Among all identified metabolites, phenolic metabolites accounted for approximately 10.18%, mainly comprising phenolic acids (169 species) and flavonoids (187 species), with only a small number of tannin metabolites (13 species) (Figure 2a).

Principal component analysis (PCA) of samples A-G, A-Z and A-S revealed PC1, PC2 and PC3 accounted for 37.63%, 16.62% and 11.49% of total variance, respectively (Figure 2b and Figure S3). The three groups presented clear intergroup separation and tight intragroup clustering, indicative of pronounced metabolic divergence. A-G and A-Z were distinguishable only along PC3, suggesting minor metabolomic variations between trunks and branches of *L. gmelinii*. In contrast, both A-G and A-Z were distinctly separated from A-S along PC1, indicating large metabolic differences between shoot stems and trunks/branches. OPLS-DA further confirmed the metabolic discrepancies among the three groups (Figure S4).

#### 2.2.2. Screening and Analysis of Differential Metabolites

Analysis of differential metabolites (DMs) across the three comparison groups (A-Z vs. A-G, A-S vs. A-G, and A-S vs. A-Z) demonstrated that the classes of phenolic acids and flavonoids occupied higher proportions in shoot stems relative to trunks and branches of *L. gmelinii*.

Volcano plots of DMs demonstrated that among the three groups, A-S vs. A-G and A-S vs. A-Z possessed abundant significant DMs with scattered fold change (FC) values: 1865 DMs (919 upregulated and 946 downregulated) and 1869 (1140 upregulated and 729 downregulated), respectively (Figure 2c). The classes of flavonoids (5~6%) and phenolic acids (5%) constituted a relatively large fraction of these identified DMs (Figure 2d). In contrast, A-Z vs. A-G contained fewer DMs (1234 in total, including 926 upregulated and 308 downregulated). Its FC values were intensively distributed near the coordinate origin, indicating a high degree of metabolic similarity between A-Z and A-G. In this group, the classes of lipids (8%) and terpenoids (6%) accounted for relatively higher proportions, whereas flavonoids (4%) and phenolic acids (4%) accounted for lower proportions.

Venn analysis revealed that 1366 DMs were commonly shared between A-S vs. A-Z and A-S vs. A-G (Figure 2e). Among these DMs, 467 metabolites showed significant discrepancies across A-S, A-Z, and A-G and were categorized as group 1 (tissue-conserved DMs). These metabolites represent core metabolic variations conserved across different stem tissues, which reflected key metabolic traits that collectively regulate tissue differentiation. A total of 899 metabolites presented no significant differences between A-Z and A-G but differed significantly between A-S and A-Z/A-G, which were classified as group 2 (shoot-specific DMs). These metabolites represent the metabolic traits specific to shoot tissues and were closely associated with the physiological activities and biological functions of shoots. In comparison, group 1 shared a highly similar metabolite class composition with the A-Z vs. A-G comparison group. In contrast, group 2 exhibited higher relative proportions of phenolic acids (5%), flavonoids (7%), organic acids (17%), and benzene and its substituted derivatives (11%) than group 1 and A-Z vs. A-G (Figure 2f).

#### 2.2.3. Differential Phenolic Acid Metabolites and Functional Enrichment Analysis

**Figure 3:**
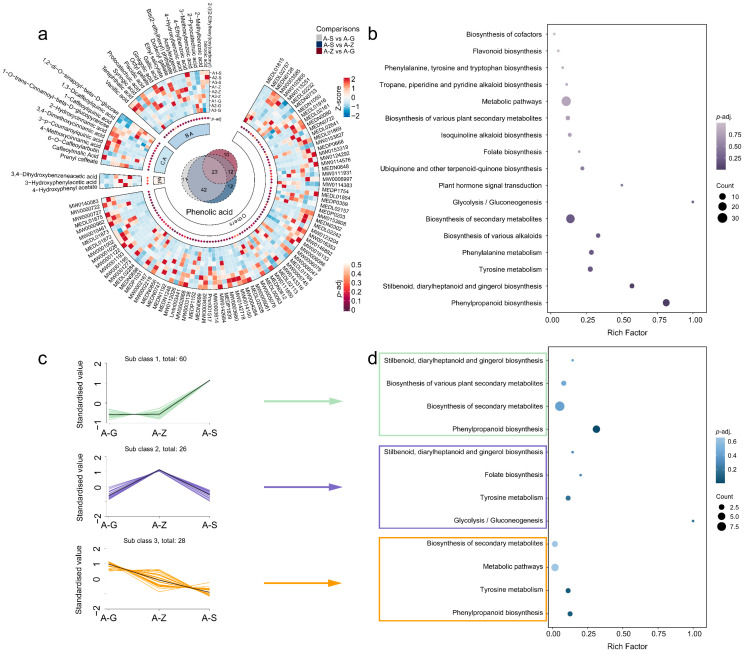
Phenolic acid divergence and functional analysis of shoots, branches and trunks of *L. gmelinii*. (**a**) Heatmap of DPMs based on Z-score normalization (BA: benzoic acid-type phenolic acids; CA: cinnamic acid-type phenolic acids; PA: phenylacetic acid-type phenolic acids); (**b**) KEGG enrichment results of all DPMs; (**c**) expression clustering analysis of all DPMs; (**d**) top 4 KEGG enrichment pathways for each subclass.

A total of 171 phenolic acid metabolites were identified across three stem tissues, among which 119 were screened as differential phenolic acid metabolites (DPMs). These DPMs consisted of 18 benzoic acid-type, three phenylacetic acid-type, and 11 cinnamic acid-type metabolites (Figure 3a and Figure S5). The remaining DPMs occurred in the form of diverse conjugated derivatives, including amides, esters, and glycosides (Table S1). Compared with A-Z vs. A-G, A-S vs. A-Z and A-S vs. A-G contained more DPMs and shared DPMs. Most DPMs exhibited relatively high levels in A-S and low levels in both A-Z and A-G.

KEGG enrichment analysis of the 119 DPMs showed that all DPMs in *L. gmelinii* were enriched in the primary pathway Metabolism. These enriched pathways covered the core pathway for phenolic acid biosynthesis (phenylpropanoid biosynthesis, ko00940), upstream carbon source and precursor metabolic pathways (glycolysis/gluconeogenesis, ko00010; tyrosine metabolism, ko00350), major secondary metabolic pathways (biosynthesis of secondary metabolites, ko01110; biosynthesis of various plant secondary metabolites, ko00999), and multiple auxiliary metabolic pathways (Figure 3b).

Based on relative abundance, the identified DPMs were categorized into three major subclasses. Subclass 1 consisted of 60 DPMs with higher levels in A-S, suggesting active biosynthesis and accumulation of phenolic acids within shoot stems. Subclass 2 contained 26 DPMs with higher levels in A-Z, reflecting the inherent phenolic acid metabolic traits of branches. Subclass 3 comprised 28 DPMs with higher levels in A-G, representing the phenolic acid metabolic profile of trunks (Figure 3c). In addition, five DPMs could not be assigned to any of the three subclasses (Table S2).

KEGG enrichment analysis of the three main subclasses further revealed that Subclass 1 was significantly enriched exclusively in phenylpropanoid biosynthesis (ko00940). By contrast, Subclass 2 and Subclass 3 failed to obtain statistically significant enrichment. Subclass 2 was mainly enriched in glycolysis/gluconeogenesis (ko00010), tyrosine metabolism (ko00350), folate biosynthesis (ko00790), and stilbenoid, diarylheptanoid and gingerol biosynthesis (ko00945). Subclass 3 was primarily enriched in tyrosine metabolism (ko00350) and phenylpropanoid biosynthesis (ko00940) (Figure 3d).

### 2.3. Determination of the Effects of DPMs on N. laricinum

#### 2.3.1. Analysis of the Relative Contents of Eight Typical DPMs

To clarify the effects of phenolic acid metabolites on *N. laricinum*, eight representative phenolic acid metabolites were screened from the 119 identified DPMs, including four benzoic acid-type compounds (VA, syringic acid, PA, gallic acid), one phenylacetic acid-type compound (3,4-dihydroxybenzeneacetic acid), two cinnamic acid-type compounds (2-hydroxycinnamic acid, 3,4-dimethoxycinnamic acid), and one phenolic acid derivative (chlorogenic acid).

**Figure 4:**
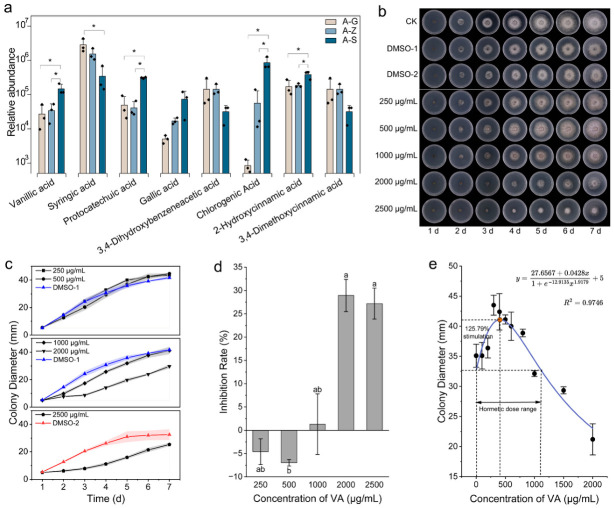
Analysis of the effects of phenolic acids on *N. laricinum*. (**a**) Comparison of the relative abundances of eight typical phenolic acids (* *p* < 0.05); (**b**) effects of VA at different concentrations on *N. laricinum*; (**c**) effects of VA at different concentrations on the growth diameter of *N. laricinum*; (**d**) inhibition rate of VA at different concentrations on *N. laricinum* at 7 d (Different lowercase letters denote significant differences at *p* < 0.05; identical letters mean no significant difference); (**e**) dose–response fitting curve between VA concentration and colony diameter of *N. laricinum*.

Previous analysis revealed that most phenolic acid metabolites presented higher levels in the shoot stems but lower levels in branches and trunks. Four of these eight DPMs (VA, PA, chlorogenic acid, 2-hydroxycinnamic acid) accumulated at significantly higher levels in A-S compared with A-G and A-Z (*p* < 0.05), which was consistent with the overall accumulation pattern of phenolic acid metabolites (Figure 4a). Most phenolic acids shared a conserved benzoic acid skeleton. Accordingly, two typical benzoic acid-type metabolites (VA and PA) were selected to investigate their biological effects on *N. laricinum*.

#### 2.3.2. Effects of VA and PA on *N. laricinum*

Bioassays of VA and PA revealed that these two phenolic acids exerted divergent biological effects on *N. laricinum*. At the identical concentration of 2000 μg/mL, PA yielded an extremely low inhibitory rate of merely 1.80% against *N. laricinum*, while VA produced a prominent inhibitory effect, reaching 56.49% (Figure S6).

#### 2.3.3. Analysis of the Dose–Response Relationship of VA on *N. laricinum*

Five concentration gradients of VA were established to preliminarily assess its dose–response relationship on *N. laricinum*. The results showed that VA promoted the growth of *N. laricinum* at low concentrations, while inhibiting growth at high concentrations (Figure 4b,c). Within the tested concentration range, the maximum growth promotion rate reached 6.99%, and the maximum inhibition rate was 28.94% (Figure 4d).

To further identify the threshold concentrations of VA for growth promotion and inhibition on *N. laricinum*, 11 refined concentration gradients were established, with observations recorded after 7 days. All data were fitted using Model (2). The fitted model for growth promotion was formulated as: $y=\frac{27.6567+0.0428x}{1+e^{-12.9135}x^{1.9179}}+5$ (adjusted *R*^2^ = 0.9746, Reduced Chi-Sqr = 0.9104) (Figure 4e and Figure S7, Table S3). Based on the fitted curve, VA promoted the growth of *N. laricinum* at concentrations ranging from 2.00 μg/mL to 1117.12 μg/mL, with the maximum growth-promoting effect observed at 410.41 μg/mL (125.79% relative to the control). When the concentration exceeded the no observed adverse effect level (1117.12 μg/mL), VA began to inhibit the growth of *N. laricinum*.

#### 2.3.4. Cell Membrane Permeability in *N. laricinum* Mycelia After VA Treatment

**Figure 5:**
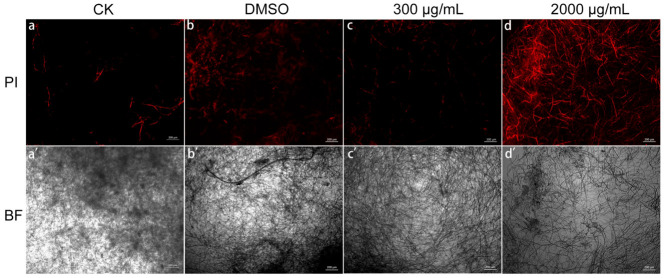
PI staining results of *N. laricinum* mycelia treated with different concentrations of VA. (**a**–**d**) PI fluorescence images; (**a′**–**d′**) corresponding bright-field (BF) micrographs. (**a**,**a′**) CK group; (**b**,**b′**) DMSO solvent control; (**c**,**c′**) treatment with 300 μg/mL VA; (**d**,**d′**) treatment with 2000 μg/mL VA. Scale bars represent 200 μm.

The results revealed that mycelia in the CK group retained intact cell structures, with barely any red fluorescence detected (Figure 5). Relative to the DMSO-treated group, mycelia exposed to 300 μg/mL VA exhibited reduced cell membrane permeability, and only a small fraction of cells emitted faint fluorescence. When the VA concentration increased to 2000 μg/mL, fluorescence intensity was significantly enhanced. This finding indicated that high-concentration VA inflicted prominent damage to the cell membrane of *N. laricinum*.

### 2.4. Mechanistic Analysis of the Biphasic Dose–Response of VA on N. laricinum

#### 2.4.1. Transcriptome Quality Assessment

To explore the molecular mechanisms underlying the growth-promoting and growth-inhibiting effects of VA on *N. laricinum*, transcriptomes of *N. laricinum* exposed to low-concentration VA (300 μg/mL, VA-L) and high-concentration VA (2000 μg/mL, VA-H) were sequenced and analyzed. Transcriptomes of samples treated with 1% DMSO were set as the control group (DMSO). QC was performed on all raw sequencing data before downstream analyses. After filtering out low-quality reads from raw data of the three sample groups (DMSO, VA-L, VA-H), 40.57 M, 43.3 M, and 43.4 M clean reads were obtained, respectively. All samples had Q20 and Q30 values of all samples exceeding 90%, which confirmed the high quality of the transcriptome sequencing data (Table S4). All genes were annotated using multiple public databases, including SwissProt, Pfam, NR, GO, and KEGG (Table S5). The expression patterns of the five differentially expressed genes (DEGs) validated via real-time quantitative PCR analysis (RT-qPCR) were generally consistent with transcriptome results, confirming the reliability of the transcriptome data (Figure S8).

#### 2.4.2. Transcriptomic Analysis of *N. laricinum* Following Low-Concentration VA Treatment

**Figure 6:**
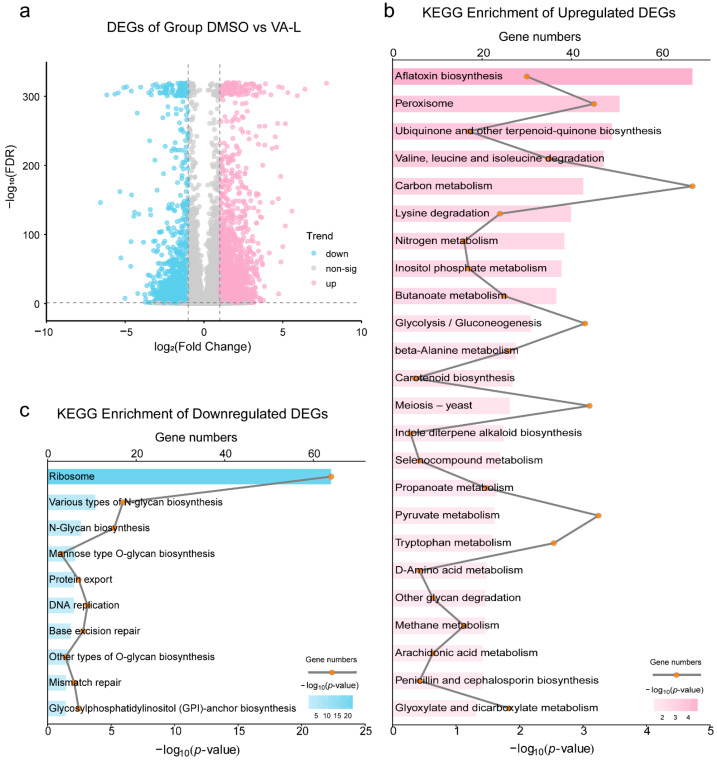
Transcriptomic analysis of *N. laricinum* mycelia after low-concentration VA treatment. (**a**) Volcano plot of DEGs after low-concentration treatment (screened with |log_2_FC| > 1 and FDR < 0.05); (**b**) significantly enriched KEGG pathways of upregulated DEGs; (**c**) significantly enriched KEGG pathways of downregulated DEGs.

A total of 3576 DEGs were identified in the DMSO vs. VA-L comparison group, including 2315 upregulated and 1261 downregulated genes (Figure 6a).

KEGG enrichment analysis for upregulated genes revealed that these genes were annotated to 132 unique biological pathways following low-concentration VA treatment relative to the DMSO group. Significant enrichment was observed in multiple functional categories, including strain virulence-related pathways (aflatoxin biosynthesis, indole diterpene alkaloid biosynthesis, penicillin and cephalosporin biosynthesis), pathways associated with fatty acid oxidation and reactive oxygen species metabolism (peroxisome), membrane lipid metabolism pathways (arachidonic acid metabolism, ubiquinone and other terpenoid-quinone biosynthesis), central carbon and energy metabolism pathways (carbon metabolism, glycolysis/gluconeogenesis, pyruvate metabolism, glyoxylate and dicarboxylate metabolism, propanoate metabolism, butanoate metabolism), and amino acid metabolism pathways (valine, leucine and isoleucine degradation, lysine degradation, tryptophan metabolism, beta-alanine metabolism, D-amino acid metabolism) (Figure 6b).

For downregulated genes, KEGG enrichment analysis indicated that these genes were mapped to 121 functional pathways under low-concentration VA treatment compared to the DMSO group. Among them, genetic information processing pathways (ribosome, DNA replication, base excision repair, mismatch repair), glycosylation modification and glycan chain biosynthesis pathways (glycosylphosphatidylinositol (GPI) anchor biosynthesis, multiple types of N-glycan biosynthesis and O-glycan biosynthesis), and protein synthesis and secretion pathways (ribosome, protein export) were enriched significantly (Figure 6c).

#### 2.4.3. Transcriptomic Analysis of *N. laricinum* Following High-Concentration VA Treatment

**Figure 7:**
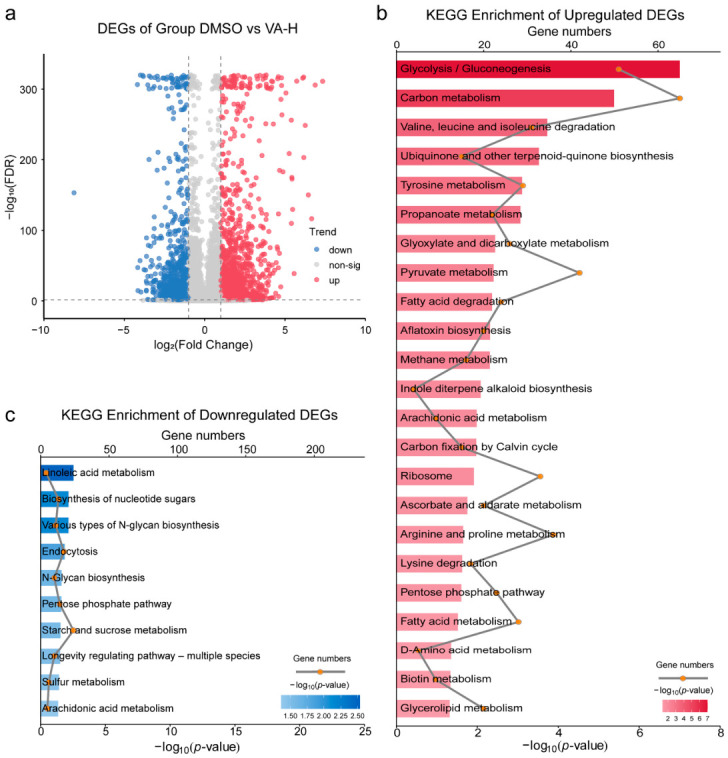
Transcriptomic analysis of *N. laricinum* mycelia after high-concentration VA treatment. (**a**) Volcano plot of DEGs after high-concentration treatment (screened with |log_2_FC| > 1 and FDR < 0.05); (**b**) significantly enriched KEGG pathways of upregulated DEGs; (**c**) significantly enriched KEGG pathways of downregulated DEGs.

A total of 2750 DEGs were identified in the DMSO vs. VA-H comparison group, including 1606 upregulated and 1144 downregulated genes (Figure 7a).

KEGG enrichment analysis for upregulated genes revealed that these genes were annotated to 122 unique pathways following high-concentration VA treatment relative to the DMSO group. Significant enrichment was detected across multiple functional modules, including strain virulence-related pathways (indole diterpene alkaloid biosynthesis, aflatoxin biosynthesis), membrane lipid metabolism pathways (glycerolipid metabolism, fatty acid metabolism, fatty acid degradation, arachidonic acid metabolism and ubiquinone and other terpenoid-quinone biosynthesis), central carbon and energy metabolism pathways (carbon metabolism, glycolysis/gluconeogenesis, pyruvate metabolism, glyoxylate and dicarboxylate metabolism, pentose phosphate pathway, Calvin cycle for carbon fixation and propanoate metabolism), amino acid metabolism pathways (lysine, valine, leucine and isoleucine degradation, tyrosine degradation, arginine and proline metabolism, D-amino acid metabolism), and protein synthesis pathway (ribosome) (Figure 7b).

For downregulated genes, KEGG enrichment analysis indicated that these genes were mapped to 123 pathways under high-concentration VA treatment compared to the DMSO group. Among them, membrane lipid metabolism-related pathways (linoleic acid metabolism, arachidonic acid metabolism), carbohydrate metabolism and glycosylation pathways (pentose phosphate pathway, starch and sucrose metabolism, nucleotide sugar biosynthesis, N-glycan biosynthesis and various types of N-glycan biosynthesis), substance transport pathway (endocytosis), and cellular homeostasis pathways (longevity regulating pathway-multiple species, sulfur metabolism) were enriched significantly (Figure 7c).

#### 2.4.4. Transcriptomic Analysis of *N. laricinum* Between VA-L and VA-H

**Figure 8:**
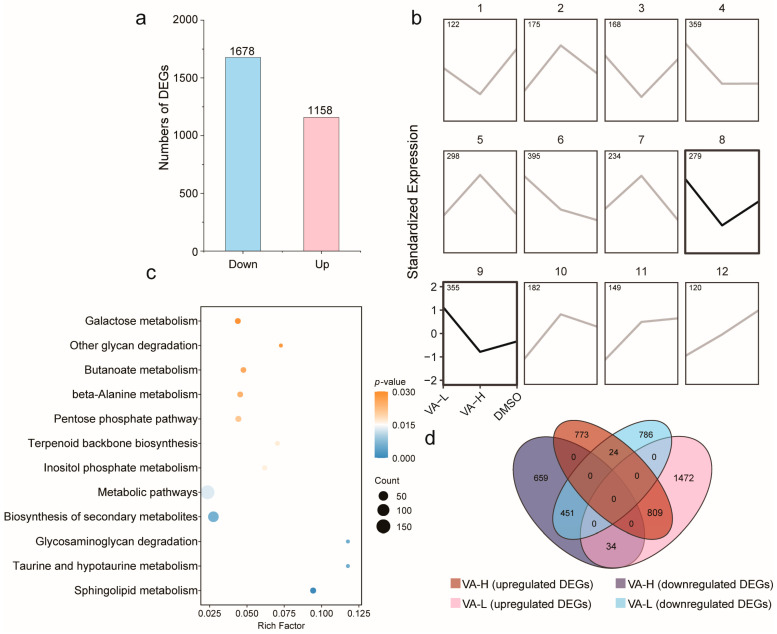
Transcriptomic analysis of VA-L vs. VA-H. (**a**) Number of DEGs between VA-L and VA-H; (**b**) Mfuzz-based clustering of gene expression patterns (Each subgraph corresponds to an individual subclass, with the number displayed in the upper-left corner denoting the total DEG number within each subclass); (**c**) significantly enriched KEGG pathways associated with Subclass 8 and Subclass 9; (**d**) overlapping DEGs shared by the DMSO vs. VA-L and DMSO vs. VA-H comparison groups.

A total of 2836 DEGs were identified in the VA-L vs. VA-H group, including 1158 upregulated and 1678 downregulated genes (Figure 8a).

Mfuzz trend clustering classified all DEGs into 12 subclasses. Subclass 8 and 9 exhibited expression patterns that matched the phenotypic traits of *N. laricinum* across different treatments (Figure 8b). Relative to the DMSO group, genes within these two subclasses were upregulated under VA-L treatment but downregulated under VA-H treatment, with significant expression discrepancies detected between the two dosage groups. These candidate genes were considered important regulators responsible for strain responses to VA exposure. KEGG enrichment analysis was performed on the 634 DEGs retrieved from Subclass 8 and Subclass 9. The analysis revealed significant enrichment in functional pathways involving energy metabolism, signal transduction, stress tolerance and cell wall structural modulation (*p* < 0.05) (Figure 8c). Venn analysis between the DMSO vs. VA-L and DMSO vs. VA-H datasets further identified 34 overlapping DEGs that were significantly upregulated under low-concentration VA and downregulated under high-concentration VA relative to the DMSO group (Figure 8d). Of these overlapping DEGs, 33 members were assigned to Subclass 8 and Subclass 9. These 33 genes were annotated to 24 KEGG pathways, among which four exhibited statistically significant enrichment: carbon fixation by the Calvin cycle (ko00710), pentose phosphate pathway (ko00030), D-amino acid metabolism (ko00470), and sulfur relay system (ko04122) (*p* < 0.05) (Figure S9).

#### 2.4.5. Integrated Analysis of the Biphasic Dose–Response Mechanism

Following exposure to low- and high-concentration VA, *N. laricinum* displayed a typical biphasic dose–response phenotype, namely promotion at low concentrations and growth inhibition at high concentrations. Nevertheless, partial similarities in gene expression profiles and functional enrichment were observed between the two dosage groups (Figure 8d). Transcriptomic comparisons indicated that upregulated DEGs from both VA-L and VA-H groups were significantly enriched in pathways associated with phenolic acid degradation, utilization and transportation. These pathways included carbon metabolism, fatty acid metabolism, fatty acid degradation, ABC transporters, and peroxisome.

Notably, the enrichment patterns of the peroxisome pathway differed substantially between the two groups. Within this pathway, enriched upregulated DEGs were mainly annotated to 27 proteins via the swissprot database. Most corresponding genes participated in aromatic compound degradation and energy metabolism, while a subset maintained intracellular redox homeostasis. A total of 13 proteins encoded by overlapping upregulated genes were shared across the two groups. These candidates included hxnS, srdG, patO, SorD, MSY001_2108, ARMGADRAFT_1018421 and DAO1. The remaining proteins were functionally linked to aromatic compound degradation, energy metabolism and oxidative stress responses, such as SOD1, SOD2, sodC, DAO1 and MSY001_2108 (Figure S10 and Table S6).

The VA-L group possessed a larger number of annotated proteins associated with aromatic compound degradation and energy metabolism, including POT1, POX3, imqH and fap2 (Table S7). Additionally, upregulated DEGs identified in the VA-L group were enriched in membrane lipid metabolism pathways, such as arachidonic acid metabolism.

Conversely, the quantity of annotated proteins involved in aromatic compound degradation and energy metabolism decreased markedly in the VA-H group (Table S8). Genes encoding SODC and SOD2 proteins, alongside genes associated with MFS transporters, exhibited elevated expression levels. Furthermore, downregulated DEGs from the VA-H group showed significant enrichment in multiple functional pathways, including arachidonic acid metabolism, linoleic acid metabolism, and endocytosis.

## 3. Discussion

Larch shoot blight is an insidious, rapidly spreading and highly destructive fungal disease that infects the stem tissues of larches [10,29]. In recent years, research on the interaction between larch and *N. laricinum* has achieved some progress. Studies on their interaction mechanism have revealed that *N. laricinum* infects larch by first degrading host cell walls and then inhibiting the host antioxidant defense system [30]. In addition, larch produces a variety of secondary metabolites, such as farrerol and evodine, which play an important role in resisting infection by *N. laricinum* [1,8]. However, phenolic acids, a group of secondary metabolites closely linked to plant defense, have not yet been thoroughly investigated for their roles in the interaction between larch and *N. laricinum*. This study found that phenolic acid metabolic profiles in different stem tissues of *L*. *gmelinii* exhibited significant tissue heterogeneity. Among these metabolites, VA, a typical benzoic acid secondary metabolite, exerted a biphasic dose–response on *N. laricinum*; this effect was affected by genes involved in lipid metabolism, carbon metabolism and cell repair. Furthermore, the geographic distribution of larch shoot blight was characterized, which matched the predicted potential suitable habitats of the pathogen [31].

### 3.1. Phenolic Acid Metabolism Analysis in Larch Stem Tissues

Plants have multi-layered defense responses to counteract pathogen infection. The synthesis and accumulation of secondary metabolites, including phenolic acids, constitute a core defensive strategy that mediates plant disease resistance and inhibits pathogen colonization and spread [11,32,33,34]. Numerous studies have demonstrated that phenolic acid levels in plants are positively correlated with plant disease resistance. For example, the phenolic acid level in disease-resistant peanut seeds correlates significantly and positively with resistance to *Aspergillus flavus* infection [35]; the basal levels of phenolic acids and flavonoids in Dawei, a *Corylus* cultivar resistant to *Botrytis cinerea*, are markedly higher than those in susceptible cultivars, including Qiuxiang [36].

This study revealed that the relative levels of multiple phenolic acids in larch shoots were significantly higher than those in branches and trunks. Shoots are tender tissues with incompletely lignified mechanical structures, rendering them highly vulnerable to invasion. Therefore, chemical defenses are more critical for young shoots than physical barriers. Compounds including phenolic acids and flavonoids serve as core substances for plant chemical defenses, which can directly suppress pathogen infection or repel pests [37]. In addition, phenolic acids and flavonoids act as precursors for lignin biosynthesis [38,39]. In this study, the proportions and enrichment trends of phenolic acids and flavonoids in shoots were highly consistent with this physiological trait. In contrast, branches and trunks shared highly similar metabolic profiles. Their differences mainly resided in structural and transport-related metabolic pathways (e.g., lipids and terpenoids), while these tissues exhibited lower relative levels of most phenolic acids. This result aligns well with the inherent functional traits of mature branches and trunks, which primarily assume nutrient transport and mechanical support. These results are consistent with the consensus on plant disease resistance mechanisms. These findings clarified the differences in phenolic acid metabolism in *L. gmelinii* stems and provided a dataset for further exploring the molecular mechanisms underlying phenolic acid metabolism during the interaction between *L. gmelinii* and *N. laricinum*.

However, despite pronounced tissue heterogeneity in phenolic acid metabolism levels across *L. gmelinii* stem tissues, *N. laricinum* still preferentially infects larch shoots. The inherent correlation between these two factors, along with the specific functions and regulatory mechanisms of endogenous phenolic acids in shoots on this fungal pathogen, requires further in-depth investigation.

### 3.2. Effects and Mechanistic Analysis of VA on N. laricinum

Although existing studies have confirmed that phenolic compound levels are closely associated with larch resistance to larch shoot blight, and phenolic acid metabolites have also been reported to possess antifungal activity, different phenolic acid types and doses exert distinct effects on various pathogenic fungi [40,41]. In this study, VA identified in *L. gmelinii* exhibited a biphasic dose–response on *N. laricinum*. Although this finding differs from the commonly observed monophasic dose–response relationship, such a phenomenon is not unique. Recent studies have validated this phenomenon, which is particularly prevalent among soil-borne pathogenic fungi such as *F. oxysporum*. For example, low-concentration syringic acid promotes mycelial growth and spore germination of *F. oxysporum* f. sp. *niveum* [22]; VA from the rhizosphere exudates of *Rehmannia glutinosa* exhibits the strongest growth-promoting effect on *F. oxysporum* [42]; and cinnamic acid, p-hydroxybenzoic acid, benzoic acid, VA and ferulic acid also exert prominent growth-stimulating effects on *F. oxysporum* f. sp. *fabae* [43]. The findings of this study indicated that apart from the aforementioned soil-borne pathogenic fungi, phenolic acids also exert dose-dependent growth-promoting effects on *N. laricinum*, a stem tissue-infecting pathogenic fungi.

Regarding the biphasic dose–response of phenolic acids, this study proposes two major potential molecular mechanisms. First, *N. laricinum* harbors metabolic pathways for phenolic acids, enabling it to degrade and utilize these compounds as carbon source. Previous studies have demonstrated that various pathogenic fungi have evolved multiple strategies to evade plant phenolic defenses, primarily including enzymatic degradation mediated by oxidoreductases, hydrolases and other enzymes, as well as the active efflux of phenolic compounds via ABC and MFS transporters [44]. In the present study, the enrichment of pathways associated with phenolic acid degradation, utilization and transport aligned well with this mechanism. This finding confirms that the ability of *N. laricinum* to degrade and utilize phenolic acids may explain why low-concentration VA failed to inhibit its mycelial growth. Second, the observed biphasic dose–response is closely correlated with the overcompensation theory. Although *N. laricinum* may degrade and utilize phenolic acids as carbon sources, carbon derived from low-concentration VA contributes negligibly to mycelial growth (VA concentrations accounted for only 0.5‰~1.4% of the glucose concentration). Therefore, phenolic acid degradation and utilization only partially accounted for the lack of an inhibitory effect of low-concentration VA on *N. laricinum*, instead of acting as the core driver of accelerated mycelial growth. Hormesis is an important theory accounting for biphasic dose responses. This theory describes that organisms exposed to low-concentration harmful stress activate compensatory repair pathways, thereby exhibiting superior physiological status, growth rate and adaptability compared with untreated control groups [45,46,47]. Previous studies have revealed that VA exerts antifungal activity by disrupting the cell membranes [15]. In this study, cell membrane permeability assays further verified that VA could damage the cell membrane of *N. laricinum*, indicating that low-concentration VA imposed stress on *N. laricinum*. In the VA-L treatment group, upregulated genes were enriched in pathways associated with base excision repair, nucleotide excision repair, mismatch repair, and autophagy. Furthermore, significantly enriched pathways involving membrane lipid metabolism and virulence of pathogenic fungi, including aflatoxin, penicillin, cephalosporin and indole diterpenoid alkaloid biosynthesis, further demonstrated that *N. laricinum* may trigger stress resistance and repair responses beyond the non-stress baseline, thereby enhancing mycelial growth under low-concentration VA treatment.

In summary, this study preliminarily clarified that VA exerted a biphasic dose–response on *N. laricinum*, and analyzed the function of phenolic acid degradation, utilization, and the overcompensation effect during this process. Notably, this study found that PA, a typical antifungal phenolic acid, exerted no significant inhibitory effect on *N. laricinum*. In addition, preliminary targeted metabolomics detection of VA was conducted in *L. gmelinii*. The quantified VA concentration in shoots was 42.25 ± 7.35 μg/mL. No direct observable growth-promoting effect was detected at this concentration in practical measurements, whereas the biphasic dose–response model demonstrated that the concentration fell within the range capable of stimulating mycelial growth of *N. laricinum*. This phenomenon may result from the difficulty in identifying subtle promoting effects or inherent limitations of model fitting. These results imply that the disease resistance mechanism mediated by phenolic acid-derived secondary metabolites may be compromised. In the plant microenvironment, the regulation of pathogenic fungi by phenolic acids is generally modulated by the combined actions of multiple phenolic acid compounds. Therefore, based on the quantitative profiling of phenolic acids in larch shoots, systematically exploring the independent and synergistic regulatory effects of phenolic acids on *N. laricinum* will serve as the core direction of our future research. In addition, other metabolites in larch, such as flavonoids, as well as arabinogalactan and its related compounds that are closely associated with larch disease resistance, represent promising directions for further investigation [48].

## 4. Materials and Methods

### 4.1. Survey of the Distribution of Larch Shoot Blight

This study compiled the reported distribution data of larch shoot blight in China and generated a corresponding distribution map. The map was produced using a standard base map with the map approval number GS (2023) 2767, downloaded from the Standard Map Service website of the National Administration of Surveying, Mapping and Geoinformation (http://bzdt.ch.mnr.gov.cn/ (accessed on 3 December 2025)). No modifications were made to the original base map.

### 4.2. Collection of Larch Samples

Based on the survey results on the distribution of larch shoot blight, drone imagery was used in Harbin, Heilongjiang province, to observe larch stands from above the crowns and identify relatively healthy stands of *L. gmelinii*. Three individual *L. gmelinii* trees were randomly selected from these stands. Due to the high lignification degree and significant tissue heterogeneity in the trunk and branches of larch, the whole tissues contain large numbers of interfering substances such as dead tissues, lignin and fibers, which can hardly reflect the active metabolic level. Therefore, in this study, the metabolically active cambia in the trunks and branches were used to represent the metabolic traits of larch. All samples were collected from healthy stems with no lesions or discoloration. The stripped cambia from the trunks, cambia from the branches, and one-year-old shoot segments without needles were immediately frozen in liquid nitrogen, labeled as A-G, A-Z and A-S respectively, and stored at −80 °C for subsequent untargeted metabolomic analysis. Stem tissues collected from individual trees were defined as a single biological replicate, generating 3 replicates in total.

### 4.3. Untargeted Metabolomic Analysis

Untargeted metabolomics profiling was performed by Wuhan MetWare Biotechnology Co., Ltd., Wuhan, China. Detailed analytical procedures and QC measures are provided in Supplementary Material, Section S1.1.

Global metabolic differences across groups were evaluated using unsupervised PCA and supervised orthogonal partial least squares discriminant analysis (OPLS-DA) via the Metware Cloud (https://cloud.metware.cn (accessed on 15 September 2025)). Variable importance in projection (VIP) values for the first principal component were derived from the OPLS-DA outputs. DMs were identified based on VIP (VIP > 1) and absolute Log_2_FC (|Log_2_FC| ≥ 1.0). Identified metabolites were annotated against the Kyoto Encyclopedia of Genes and Genomes (KEGG) Compound database (http://www.kegg.jp/kegg/compound/ (accessed on 15 September 2025)) and subsequently mapped to the KEGG Pathway database (http://www.kegg.jp/kegg/pathway.html (accessed on 15 September 2025)). For heatmap visualization, relative abundance values were normalized via the Z-score method, and one-way ANOVA was applied for significance testing. KEGG enrichment and K-means clustering analyses were conducted using the Metware Cloud. Among the identified DMs, representative phenolic acids were selected for further investigation. These metabolites have a well-established research background, defined biological functions, commercially available standards, or serve as typical core products of the phenylpropane metabolic pathway.

### 4.4. Determination of the Effects of VA and PA on N. laricinum

VA and PA were purchased from Shanghai Yuanye Biotechnology Co., Ltd., Shanghai, China, and dissolved into stock solutions using DMSO and anhydrous ethanol as cosolvents, respectively. The stock solutions were added to autoclaved potato dextrose agar (PDA) medium to a final concentration of 2000 μg/mL, with the cosolvent concentration maintained at 1% (*v*/*v*). The effects of VA and PA on *N. laricinum* were evaluated via the colony diameter measurement method as described in previous studies [8]. Each treatment group consisted of 5 biological replicates. The inhibition rate was calculated according to the following Formula (1).

(1) $$Inhibition\;rate=\frac{Dc-Dt}{Dc-0.05}\times 100\%$$

In this formula, D*c* represents the colony diameter after 7 d of the control group treated only with 1% cosolvent, and D*t* represents the colony diameter after 7 d of treatment with the test compounds.

### 4.5. Determination of the Dose–Response Relationship of VA on N. laricinum

To further clarify the functional role of VA in regulating larch resistance on *N. laricinum* infection, 2 groups of in vitro antifungal assays were designed. Each concentration was tested with 5 biological replicates. One group contained 6 concentration gradients (0 μg/mL, 250 μg/mL, 500 μg/mL, 1000 μg/mL, 2000 μg/mL, 2500 μg/mL) to evaluate the dose–response relationship between VA and *N. laricinum*. The inhibition rate was calculated in accordance with the following Formula (1).

The other group contained 11 concentration gradients (0 μg/mL, 100 μg/mL, 200 μg/mL, 300 μg/mL, 400 μg/mL, 500 μg/mL, 600 μg/mL, 800 μg/mL, 1000 μg/mL, 1500 μg/mL, 2000 μg/mL) to accurately identify the thresholds for the stimulatory and inhibitory effects of VA on *N. laricinum*. The biphasic dose–response relationship of VA across different concentrations was assessed via Model (2) using unweighted fitting. This classic model is widely applied to characterize dose–response patterns with low-concentration growth stimulation [49]. In this model, *y* refers to colony diameter and *x* to VA concentration. Parameter *k* quantifies the response difference between the control group and the minimum asymptote, while *f* characterizes the magnitude of the low-concentration stimulatory effect. A non-zero *f* value indicates a significant stimulatory effect. Parameter *b* describes curve steepness, *g* acts as a position parameter determining the concentration at the peak response, and *d* stands for the minimum response asymptote of treatment groups.

(2) $$y=\frac{\left(k+fx\right)}{\left(1+e^{bg}x^{b}\right)}+d$$

### 4.6. Transcriptome Sequencing and Differential Analysis

The mycelia grown on PDA plates supplemented with 1% DMSO, 300 μg/mL and 2000 μg/mL VA for 7 d were collected, snap-frozen in liquid nitrogen, and subsequently stored at −80 °C for transcriptomic sequencing. Samples were sent to BGI (Beijing, China) for RNA extraction, library construction, transcriptome sequencing, and sequence assembly. Each treatment group consisted of 3 biological replicates. Transcriptome data were generated using the Illumina NovaSeq 6000 platform (Illumina Inc., San Diego, CA, USA), with paired-end sequencing technology employed for RNA sequencing. After data quality control, FastQC (http://www.bioinformatics.babraham.ac.uk/projects/fastqc/ (accessed on 15 October 2025)) was used to comprehensively evaluate the sequence quality. Trinity software 2.15.1 was applied to perform de novo assembly of clean reads for the acquisition of transcripts (No reference genome). All assembled unigenes were functionally annotated against the Non-Redundant (NR, http://www.ncbi.nlm.nih.gov/ (accessed on 15 October 2025)), SwissProt (http://www.expasy.ch/sprot/ (accessed on 15 October 2025)), Pfam (https://www.ebi.ac.uk/interpro/ (accessed on 15 October 2025)), Gene Ontology (GO, http://www.geneontology.org (accessed on 15 October 2025)), KEGG (http://www.genome.jp/kegg/ (accessed on 15 October 2025)) databases.

Differential expression analysis was performed using the DESeq2 package (v1.16.1) to identify DEGs. Genes with |log_2_FC| > 1 and FDR < 0.05 were defined as significantly differentially expressed. KEGG pathway enrichment analyses were performed using the dplyr, clusterProfiler, and ggplot2 packages in R software (v4.5.1).

### 4.7. Real-Time Quantitative PCR Analysis

To validate the transcriptomic sequencing results, RT-qPCR analysis was performed on the mycelia of *N. laricinum* grown for 7 d on PDA medium containing 1% DMSO, 300 μg/mL and 2000 μg/mL VA, in accordance with the methods described in the literature [50]. Five random genes were used as target genes and amplified using the primer pairs (Table S9). *V-ATP* was used as the internal control.

### 4.8. Cell Membrane Integrity Detection

Cell membrane integrity was evaluated by PI staining. Two-day-cultured mycelia were treated with 0 (1% DMSO), 300 and 2000 μg/mL VA at 25 °C for 6 h, using untreated normal mycelia as the control. Mycelia were incubated in 300 μL PI solution in the dark at 25 °C for 10 min and then washed 3 times with phosphate-buffered saline (PBS) to remove residual dye [51]. Fluorescence imaging was acquired using a Zeiss Axio Scope microscope (Carl Zeiss, Oberkochen, Germany) equipped with a 5× objective lens (total optical magnification: 50×), a 1× intermediate magnification changer, and a 488 nm filter set. Images were recorded with an Axio-cam 506 camera at an exposure of 4.6 s. All obtained images contained calibrated scale bars.

### 4.9. Data Statistical Analysis and Plotting

Data analysis was performed using Excel 2602, the Metware Cloud, the dplyr package and tidyverse package of R software 4.5.1. Graphs were plotted using the ggplot2 package, ggprism package, ggrepel package, ComplexHeatmap package, and VennDiagram package.

## 5. Conclusions

In the stem tissues of larch, phenolic acid metabolism exhibits tissue heterogeneity. Furthermore, VA, a typical benzoic acid-type phenolic acid that is significantly upregulated in shoots, may be modulated by the phenolic acid metabolic capacity and overcompensation of *N. laricinum*, and displays a distinct biphasic dose–response.

## Data Availability

The RNA sequencing data have been deposited in the NCBI Sequence Read Archive (http://www.ncbi.nlm.nih.gov/sra (accessed on 12 January 2026)) database, under the accession number (PRJNA1300662) provided. Additional data from this study are available in the Supplementary Material.

## Supplementary Materials

The following supporting information can be downloaded at: https://www.mdpi.com/article/10.3390/ijms27125159/s1.

## Abbreviations

The following abbreviations are used in this manuscript:

VA	Vanillic acid
PA	Protocatechuic acid
DMs	Differential metabolites
DPMs	Differential phenolic acid metabolites
QC	Quality control
PCA	Principal component analysis
VIP	Variable importance in projection
KEGG	Kyoto Encyclopedia of Genes and Genomes
DEGs	Differentially expressed genes
PI	Propidium iodide
PBS	Phosphate-buffered saline
OPLS-DA	Orthogonal partial least squares discriminant analysis
RT-qPCR	Real-time quantitative PCR analysis
PDA	Potato dextrose agar
