# Supplementary material for: Biphasic Dose–Response and Mechanism Analysis of Vanillic Acid from Larix gmelinii on Neofusicoccum laricinum
Source: Int J Mol Sci. 2026 Jun 6;27(12):5159. doi: 10.3390/ijms27125159 (PMC13299209; doi:10.3390/ijms27125159)
Supplement: Supplementary file 1 [file ijms-27-05159-s001.zip › ijms-4334142-supplementary.pdf]

Supplementary Material

1 Supplementary Method

S1.1 Non-targeted metabolomics determination and quality control method:

The samples were first freeze-dried and then ground into powder. For every 30 mg of sample, 1500 μL of 70% methanol aqueous solution internal standard extraction solution pre-cooled to −20°C was added, followed by thorough vortexing and centrifugation at 12000 rpm for 3 minutes. The supernatant was taken for ultra-high performance liquid chromatography-tandem mass spectrometry (UPLC-MS/MS) analysis. Pooled quality control (QC) samples were prepared by combining 10 μL of each extraction mixture. A QC sample was acquired after every 10 samples.

All samples were acquired by the LC-MS system followed machine orders. The analytical conditions were as follows, UPLC: column, Waters ACQUITY UPLC HSS T3 1.8 µm, 2.1 mm * 100 mm; column temperature, 40 °C; flow rate, 0.40 mL/min; injection volume, 4 μL; solvent system, water (0.1 % formic acid): acetonitrile (0.1 % formic acid); Sample measurements were performed with a gradient program that employed the starting conditions of 95% A, 5% B. Within 5 min, a linear gradient to 35% A, 65% B was programmed, Within 1 min, a linear gradient to 1% A,99% B was programmed, kept for 1.5 min. Subsequently, a composition of 95% A, 5.0% B was adjusted within 0.1 min and kept for 2.4 min.

The data acquisition was operated using the information-dependent acquisition (IDA) mode using Analyst TF 1.7.1 Software (Sciex, Concord, ON, Canada). The source parameters were set as follows: ion source gas 1 (GAS1),50 psi; ion source gas 2 (GAS2), 60 psi; curtain gas (CUR), 35 psi; temperature(TEM), 550 °C, or 550 °C; declustering potential (DP), 80 V, or

−80 V in positive or negative modes, respectively; and ion spray voltagefloating (ISVF), 5500 V or −4500 V in positive or negative modes, respectively. The TOF MS scan parameters were set as follows: mass range, 50–1250 Da; accumulation time, 200 ms; and dynamic background subtract, on. The product ion scan parameters were set as follows: mass range, 50–1250 Da; accumulation time, 40 ms; collision energy, 30 or −30 V in positive or negative modes, respectively; collision energy spread, 15; resolution, UNIT; charge state, 1 to 1; intensity, 100 cps; exclude isotopes within 4 Da; mass tolerance, 50 mDa; maximum number of candidate ions to monitor per cycle, 12.

The raw data of liquid chromatography-tandem mass spectrometry (LC-MS/MS) were converted to mzML format using ProteoWizard and processed with XCMS program, which mainly included peak extraction, alignment, correction, etc. The peaks after correction and screening were identified for metabolites by searching the self-built database of MetWare platform, integrating public databases, prediction databases, and using the metDNA method. The technical repeatability of metabolite extraction and detection was judged by overlapping display and analysis of the total ion current (TIC) charts from mass spectrometry detection and analysis of different quality control (QC) samples.

2 Supplementary Figures and Tables

2.1 Supplementary Figures

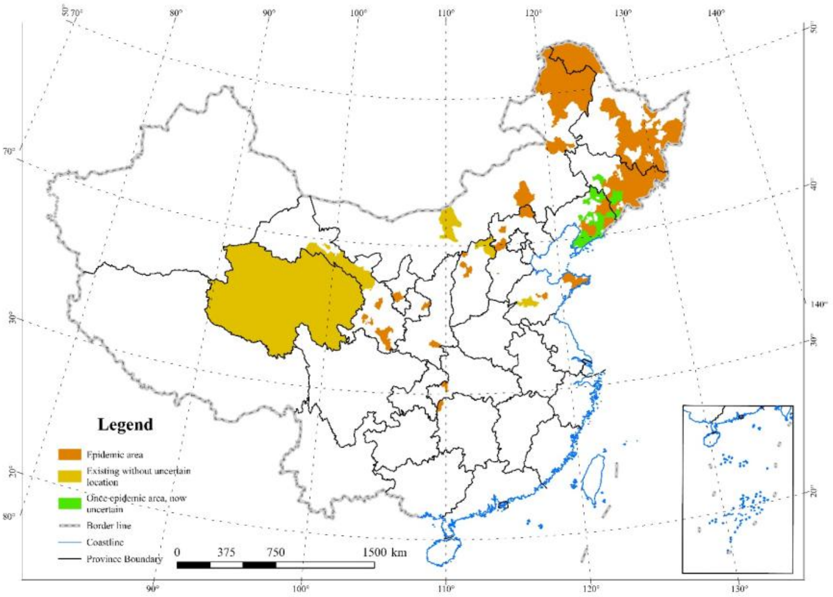

Figure S1 Reported distribution areas of larch shoot blight

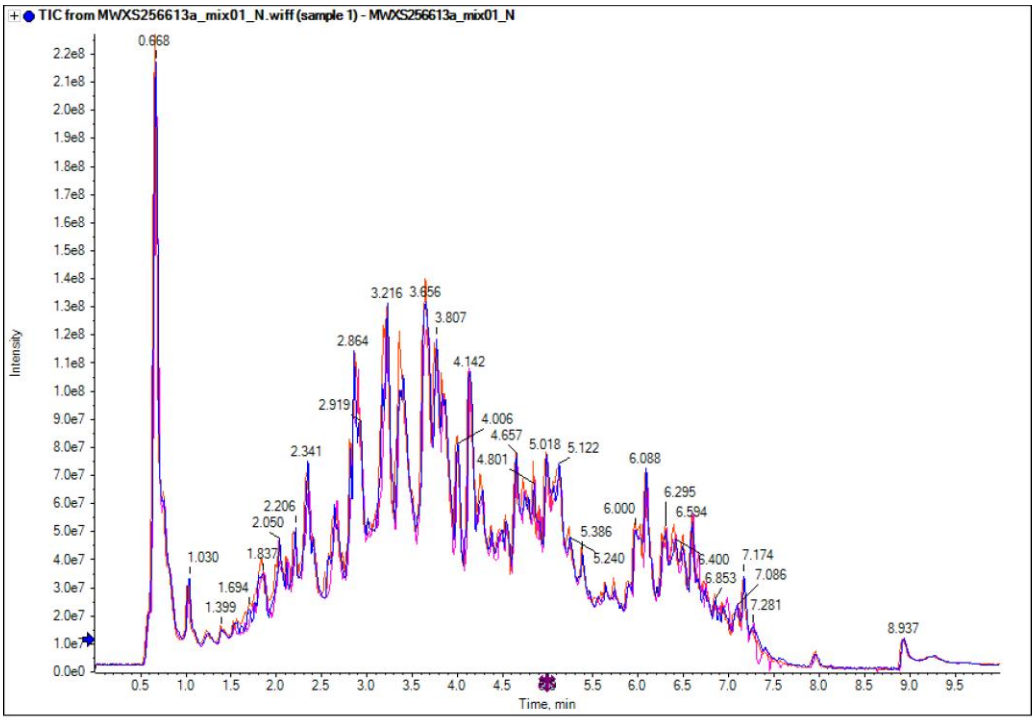

Figure S2 QC quality control chart for non-targeted metabolomics

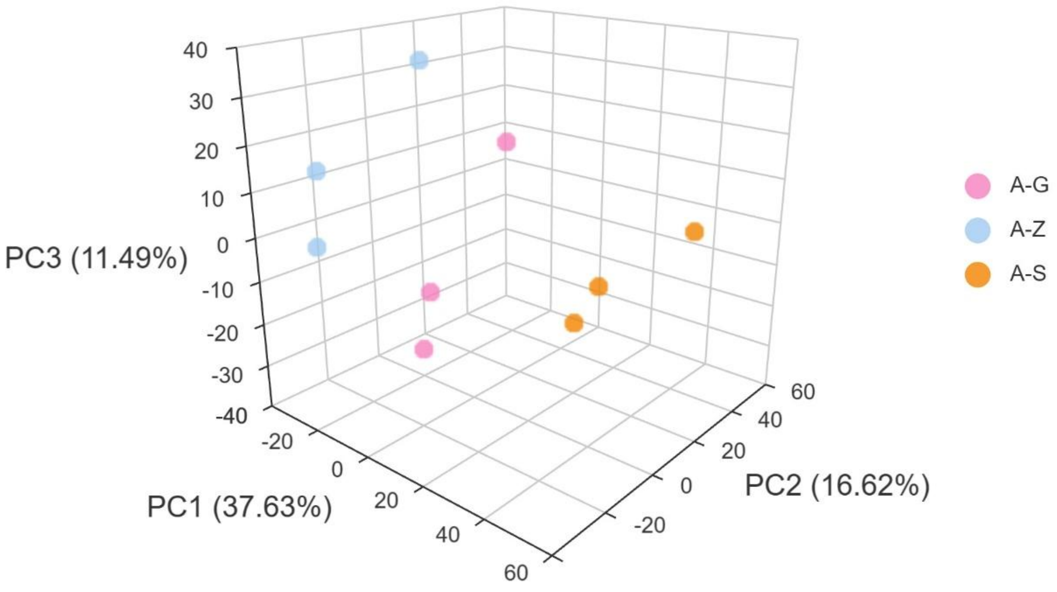

Figure S3 Three-dimensional PCA plot of non-targeted metabolomics

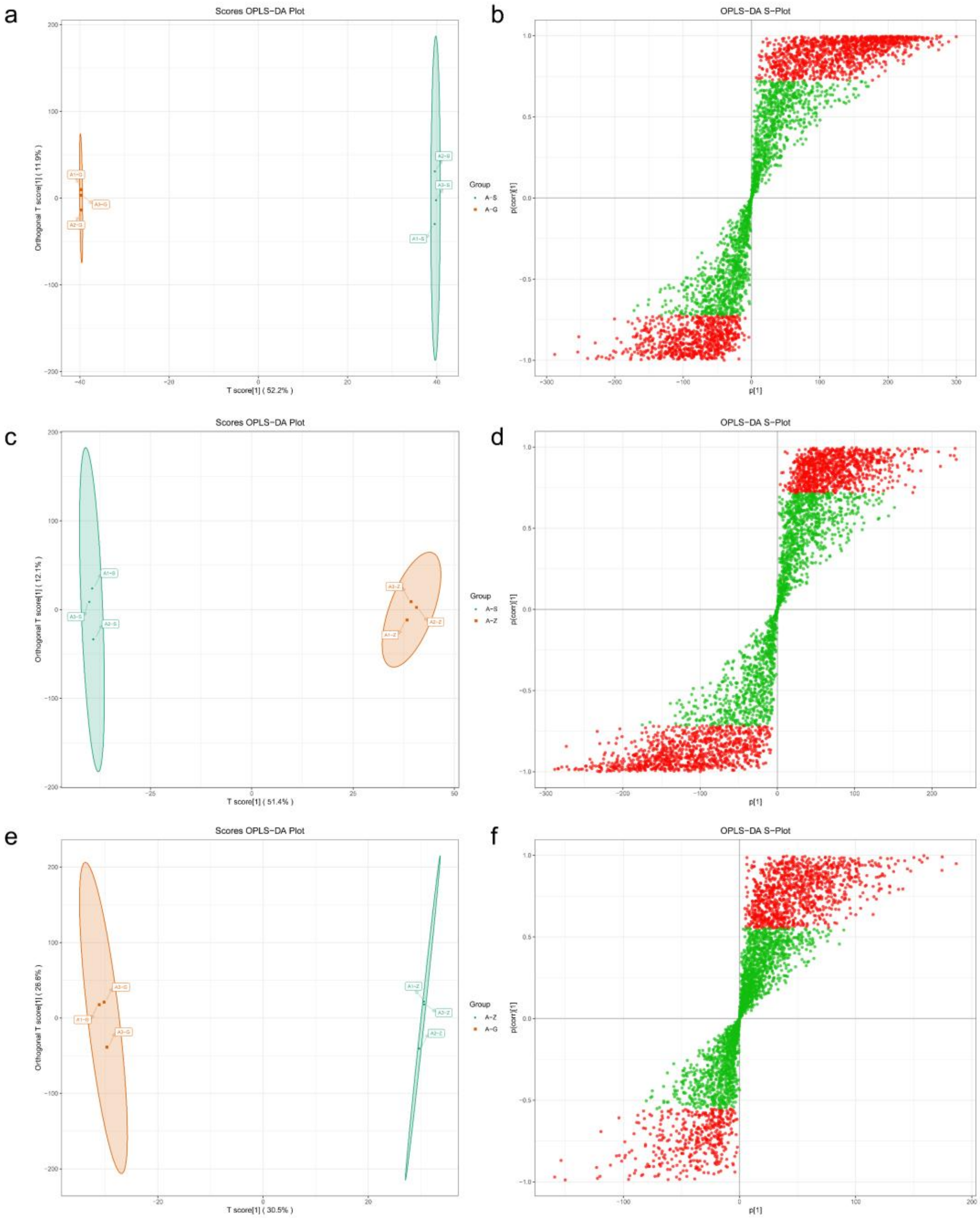

Figure S4 OPLS-DA analysis of non-targeted metabolomics. **a** OPLS-DA score plots of groups A-G and A-S; **b** OPLS-DA S-Plot of groups A-G and A-S; **c** OPLS-DA score plots of groups A-S and A-Z; **d** OPLS-DA S-Plot of groups A-S and A-Z; **e** OPLS-DA score plots of groups A-Z and A-G; f OPLS-DA S-Plot of groups A-Z and A-G.

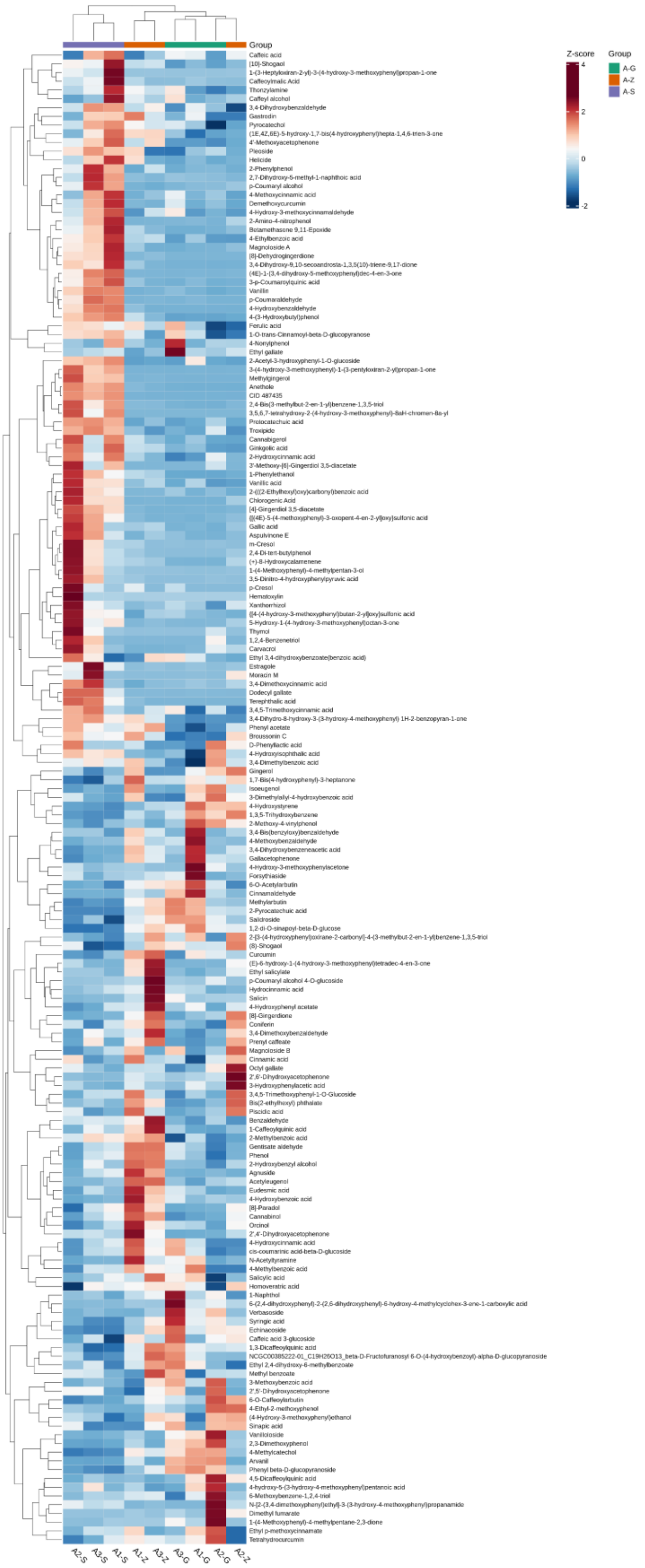

Figure S5 Cluster heatmap of 171 PMs based on Z-score normalization

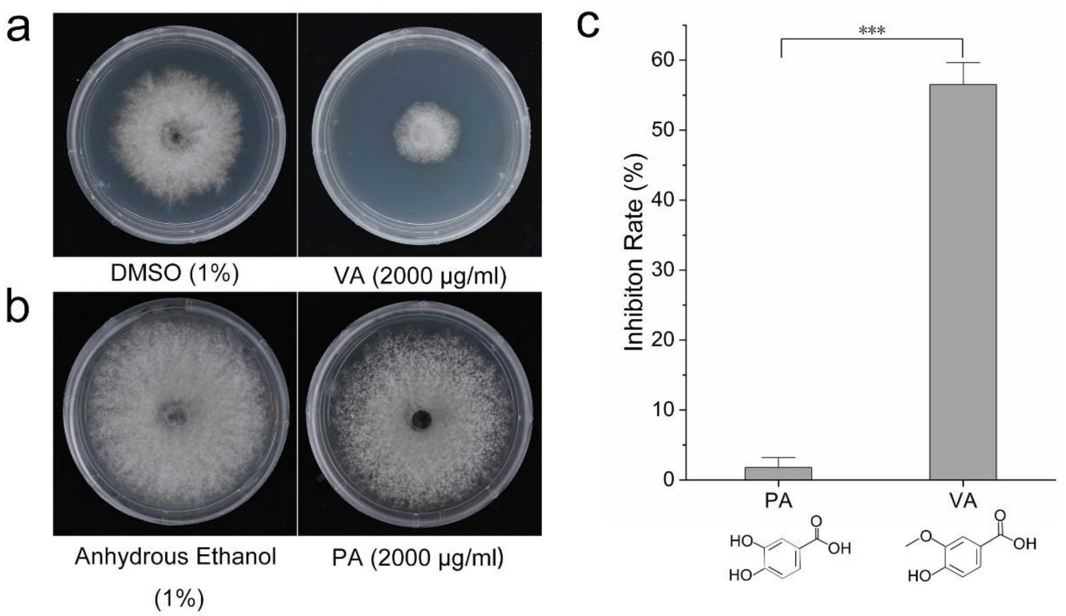

Figure S6 The effects of PA and VA on *N. laricinum*. ***$P < 0.001$

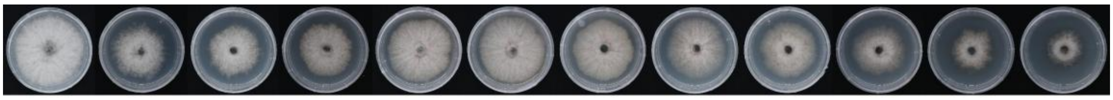

Figure S7 Colony growth status of *N. laricinum* at 11 VA concentration treatments for 7 days

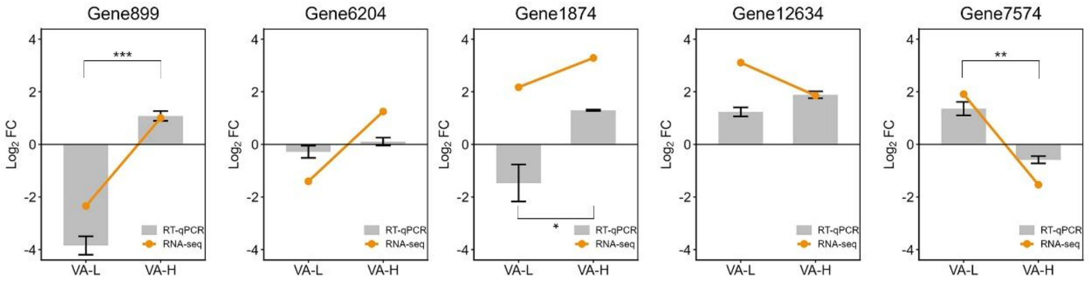

Figure S8 The relative expression levels of 5 DEGs. *$P < 0.05$, **$P < 0.01$, **$P < 0.001$

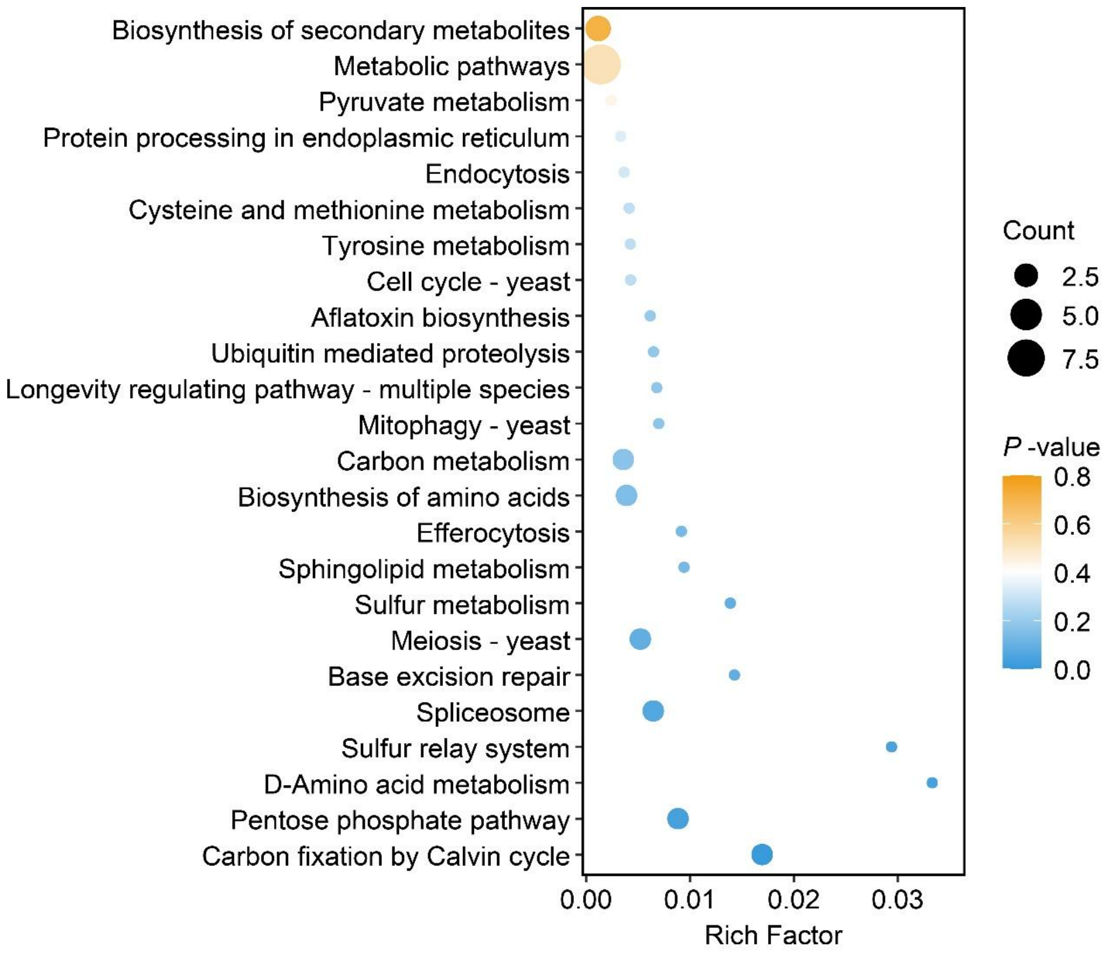

Figure S9 KEGG enrichment results of 33 DEGs

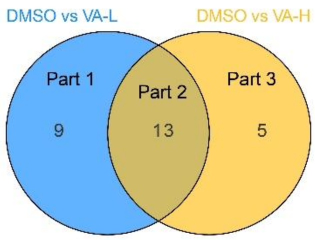

Figure S10 Venn diagram of Swissprot annotations for upregulated DEGs in the DMSO vs VA-L and DMSO vs VA-H groups

2.2 Supplementary Tables

Table S1 88 types of DPMs existing in various conjugated derivative forms such as amides, esters or glycosides

Index	Compounds
pmn001517	3,4,5-Trimethoxyphenyl-1-O-Glucoside
MW0153827	Methylarbutin
MW0153808	CID 487435
MW0153319	Magnoloside A
MW0146882	Broussonin C
MW0143316	5-Hydroxy-1-(4-hydroxy-3-methoxyphenyl)octan-3-one
MW0142718	3,5-Dinitro-4-hydroxyphenylpyruvic acid
MW0142684	3,4-Dihydroxy-9,10-secoandrosta-1,3,5(10)-triene-9,17-dione
MW0140083	(+)-8-Hydroxycalamenene
MW0124292	Hematoxylin
MW0123204	Cannabinol
MW0115251	Salidroside
MW0114576	Helicide
MW0114383	Echinacoside
MW0112026	2-[3-(4-hydroxyphenyl)oxirane-2-carbonyl]-4-(3-methylbut-2-en-1-yl)benzene-1,3,5-triol
MW0111931	Gallacetophenone
MW0111650	4'-Methoxyacetophenone
MW0102805	Thymol
MW0016353	Cannabigerol
MW0016133	Betamethasone 9,11-Epoxide
MW0014150	3'-Methoxy-[6]-Gingerdiol 3,5-diacetate
MW0010461	[8]-Dehydrogingerdione
MW0006997	Estragole
MW0006086	Aspulvinone E
MW0006079	Arvanil
MW0006047	Anethole
MW0005745	6-(2,4-dihydroxyphenyl)-2-(2,6-dihydroxyphenyl)-6-hydroxy-4-methylcyclohex-3-ene-1-carboxylic acid
MW0005076	4-hydroxy-5-(3-hydroxy-4-methoxyphenyl)pentanoic acid
MW0005061	4-Hydroxy-3-methoxyphenylacetone
MW0004284	4-(3-Hydroxybutyl)phenol
MW0003660	3,5,6,7-tetrahydroxy-2-(4-hydroxy-3-methoxyphenyl)-8aH-chromen-8a-yl
MW0003614	3,4-Dihydro-8-hydroxy-3-(3-hydroxy-4-methoxyphenyl) 1H-2-benzopyran-1-one
MW0003585	Troxipide
MW0003492	3-(4-hydroxy-3-methoxyphenyl)-1-(3-pentyloxiran-2-yl)propan-1-one
MW0003138	2-Hydroxybenzyl alcohol
MW0002668	2-Amino-4-nitrophenol

Index	Compounds
MW0002219	2,4-Bis(3-methylbut-2-en-1-yl)benzene-1,3,5-triol
MW0002167	2,3-Dimethoxyphenol
MW0001273	1,2,4-Benzenetriol
MW0001195	1-(4-Methoxyphenyl)-4-methylpentane-2,3-dione
MW0001193	1-(4-Methoxyphenyl)-4-methylpentan-3-ol
MW0001122	1-(3-Heptyloxiran-2-yl)-3-(4-hydroxy-3-methoxyphenyl)propan-1-one
MW0001026	{[4-(4-hydroxy-3-methoxyphenyl)butan-2-yl]oxy}sulfonic acid
MW0001002	{[(4E)-5-(4-methoxyphenyl)-3-oxopent-4-en-2-yl]oxy}sulfonic acid
MW0000962	[4]-Gingerdiol 3,5-diacetate
MW0000727	(E)-6-hydroxy-1-(4-hydroxy-3-methoxyphenyl)tetradec-4-en-3-one
MW0000722	(4E)-1-(3,4-dihydroxy-5-methoxyphenyl)dec-4-en-3-one
MEDP1839	3,4-Dimethoxybenzaldehyde
MEDP1754	Dimethyl fumarate
MEDP1152	2-Methoxy-4-vinylphenol
MEDP1031	1-Phenylethanol
MEDP0668	m-Cresol
MEDP0359	Curcumin
MEDP0203	Cinnamaldehyde
MEDN1248	2,7-Dihydroxy-5-methyl-1-naphthoic acid
MEDN1192	2',6'-Dihydroxyacetophenone
MEDN1050	Phenyl acetate
MEDN0856	2',4'-Dihydroxyacetophenone
MEDN0812	4-Hydroxystyrene
MEDN0747	2,4-Di-tert-butylphenol
MEDN0733	Phenyl beta-D-glucopyranoside
MEDN0722	Orcinol
MEDN0699	2-Phenylphenol
MEDN0698	1-Naphthol
MEDN0648	Gentisate aldehyde
MEDN0302	Chlorogenic Acid
MEDN0128	Vanillin
MEDN0080	p-Coumaraldehyde
MEDL02757	Vanilloloside
MEDL02641	Moracin M
MEDL02284	1,3,5-Trihydroxybenzene
MEDL02242	Carvacrol
MEDL02232	Salicin
MEDL02161	p-Coumaryl alcohol
MEDL02157	Coniferin
MEDL02113	4-Methylcatechol
MEDL02026	4-Hydroxy-3-methoxycinnamaldehyde
MEDL01916	Phenol
MEDL01875	[10]-Shogaol

Index	Compounds
MEDL01873	[8]-Gingerdione
MEDL01872	[8]-Paradol
MEDL01869	Methylgingerol
MEDL01854	Demethoxycurcumin
MEDL01815	Verbasoside
MEDL01768	6-O-Acetylarbutin
MEDL00063	4-Hydroxybenzaldehyde
Lmtn003441	2-Acetyl-3-hydroxyphenyl-1-O-glucoside

Table S2 Relative contents of 5 DPMs not clustered into the target category by K-means analysis

Compounds	A-G (log_2, Mean±SD)	A-Z (log_2, Mean±SD)	A-S (log_2, Mean±SD)
2-Methylbenzoic acid	13.35 ± 1.11	15.69 ± 0.77	15.76 ± 0.21
4'-Methoxyacetophenone	18.41 ± 0.21	19.72 ± 0.84	20.61 ± 0.42
Phenyl acetate	18.02 ± 0.70	20.22 ± 0.30	20.19 ± 0.27
3,4-Dihydro-8-hydroxy-3-(3-hydroxy-4-methoxyphenyl) 1H-2-benzopyran-1-one	16.99 ± 0.50	19.09 ± 1.10	20.39 ± 0.21
Broussonin C	17.72 ± 0.16	19.44 ± 0.25	19.40 ± 0.16

Data were log_2-transformed relative abundance; values are presented as Mean ± SD, n=3

Table S3 Parameters of the biphasic dose-response model for VA towards *N. laricinum*

Parameter	Estimate ± Standard Error
k	27.65665 ± 0.16432
f	0.04278 ± 0.00133
b	1.91787 ± 0.01104
d	5 ± 0
g	-6.73327 ± 0.01693
Reduced Chi-Sqr	0.91043
R^2	0.97462
Adjusted R^2	0.97455

Table S4 Quality statistics of filtered reads.

Sample	Total Raw Read (M)	Total Clean Read (M)	Total Clean Base (M)	Clean Read Q20 (%)	Clean Read Q30 (%)	Clean Read Ratio (%)
DMSO	42.16	40.57	6.08	96.82	90.38	96.21
VA-L	45.44	43.3	6.49	96.75	90.34	95.29
VA-H	45.44	43.4	6.51	96.94	90.73	95.52

Table S5 Gene annotation in databases

Database	Number	Percentage
Total	38511	100%
NR	27586	71.63%
Swissprot	17922	46.54%
Pfam	22086	57.35%
GO	20261	52.61%
KEGG	18971	49.26%

Table S6 Swissprot annotations and corresponding genes in Part 2 of the Venn diagram

Swissprot annotations	Gene ID
SOD1 (superoxide dismutase)	Gene4287
hxnS (Nicotinate hydroxylase)	Gene4817
ILV2 (Acetolactate synthase)	Gene1378, Gene7755
MSY001_2108 (GMC oxidoreductase family protein)	Gene10372
srdG (Epoxide hydrolase)	Gene11004, Gene26276
patO (FAD-linked oxidoreductase)	Gene11041, Gene12177, Gene27006
SPCC14G10.01 (Uncharacterized protein)	Gene11064, Gene11065, Gene12836
sodC (superoxide dismutase)	Gene12220
sorD (FAD-linked oxidoreductase)	Gene12392
cefD2 (Isopenicillin N epimerase component 2)	Gene16369, Gene16370, Gene16372, Gene16373, Gene16374, Gene16375
SOD2 (superoxide dismutase)	Gene16603
DAO1 (D-amino-acid oxidase)	Gene21614, Gene21811
ARMGADRAFT_1018421 (Short-chain dehydrogenase/reductase)	Gene22655, Gene22657

Table S7 Swissprot annotations and corresponding genes in Part 1 of the Venn diagram

Swissprot annotations	Gene ID
POX9 (Peroxisomal catalase)	Gene3233, Gene13993
imqH (Amino acid oxidase)	Gene3478, Gene3479, Gene16024, Gene24009
POT1 (3-ketoacyl-CoA thiolase, peroxisomal)	Gene7126
POX3 (Acyl-coenzyme A oxidase)	Gene8282
fap2 (L-saccharopine oxidase)	Gene6033, Gene12206
cat-1 (Catalase-1)	Gene14640, Gene16411
rhp57 (DNA repair protein)	Gene16984
GIT3 (Major glycerophosphoinositol permease)	Gene23661
ilv1 (Acetolactate synthase, mitochondrial)	Gene26714

Table S8 Swissprot annotations and corresponding genes in Part 3 of the Venn diagram

Swissprot annotations	Gene ID
MFS (Phomenoic acid biosynthesis cluster MFS-type transporter)	Gene373, Gene27626
PEX7 (Peroxisomal targeting signal 2 receptor)	Gene10972
pex1 (peroxisomal ATPase)	Gene26197
YNR064C (Uncharacterized hydrolase)	Gene26881
IDP2 (Isocitrate dehydrogenase)	Gene27574

Table S9 RT-qPCR primers used in this study.

Gene	Gene annotation	Forward primer (5'~3')	Reverse primer (5'~3')
Gene899	Cell wall protein *phiA*	ATCGGCAAGGAATCGAACTC	TGGTGAACTGGTCAGGCA
Gene6204	G protein-coupled receptor *GPR1*	AGGTCGGACCAGATACACC	AGGAGATGCGATGGAGAGAA
Gene1874	Isoeugenol monooxygenase	GCGAGCTATGTTGTGCTG	CCAGTGGTACGTCCTGTACT
Gene12634	Quinone oxidoreductase	GTACGGAGGAGAAGATTGCG	GACTCCAAGCTCGCATCAA
Gene7574	Transaldolase	GGAATCCGTACACTCGGAAC	GGATCATCCGTGCTGACATT
Reference gene	*V-ATP*	CCAACAAGTTCACGCTTGC	CACTTGATCATGGCAACAGC
